# Influence of lifestyle on suboptimal health: Insights from a national cross-sectional survey in China

**DOI:** 10.7189/jogh.13.04151

**Published:** 2023-11-17

**Authors:** Jie Wang, Yinghao Wang, Zheng Guo, Zi Lin, Xiangqian Jin, Hui Niu, Yibo Wu, Lihua Tang, Haifeng Hou

**Affiliations:** 1 School of Public Health, Shandong First Medical University & Shandong Academy of Medical Sciences, Jinan, China; 2 Division of Epidemiology, Department of Medicine, Vanderbilt Epidemiology Center, Vanderbilt University Medical Center, Nashville, Tennessee; 3 School of Medical and Health Sciences, Edith Cowan University, Perth, Australia; 4 Taian Maternity and Child Health Hospital, Taian, China; 5 School of Public Health, Peking University, Beijing, China; 6 Department of Blood Transfusion, The Affiliated Taian City Central Hospital of Qingdao University, Taian, China; 7 School of Public Health and The Second Affiliated Hospital of Shandong First Medical University, Taian, China

## Abstract

**Background:**

Suboptimal health status (SHS) is a non-clinical or pre-disease state between optimal/ideal health and disease. While its etiology remains unclear, lifestyle is considered one of the most important risk factors. We aimed to examine the effects of lifestyles on SHS through a nationwide survey in China.

**Methods:**

We conducted a cross-sectional survey in 148 cities across China between 20 June and 31 August 2022, on 30 505 participants from rural and urban communities gathered through stratified quota sampling. We measured SHS with the Short-Form Suboptimal Health Status Questionnaire (SHSQ-SF). We gathered information on participants’ lifestyles (ie, smoking, alcohol consumption, breakfast habits, weekly food delivery frequency, intermittent fasting, sleep duration and physical activities) through face-to-face interview. We determined the relationship between lifestyle and SHS logistic regression analysis by based on odds ratios (ORs) and 95% confidence intervals (CIs).

**Results:**

We included 22 897 participants (female: 13 056, male: 9841), 12 108 (52.88%) of whom reported exposure to SHS. After adjusting for demographic characteristics, individuals who currently smoked (OR = 1.165; 95% CI = 1.058-1.283) and those who drank alcohol (OR = 1.483; 95% CI = 1.377.1.596) were at a higher risk of SHS than those who have never done either. In a dose-response way, takeaway food consumption was associated with a higher risk of SHS, while increased frequency of breakfast and mild-intensity exercise conversely reduced said risk. Individuals with shorter sleep duration had a higher risk of SHS when compared to those who slept for more than seven hours per day.

**Conclusions:**

We observed a relatively high prevalence of SHS across China, highlighting the importance of lifestyle in health promotion. Specifically, adopting healthy dietary habits, engaging in regular physical activity, and ensuring high-quality sleep are key in preventing SHS.

**Registration:**

Chinese Clinical Trial Registry (ChiCTR2200061046).

Suboptimal health status (SHS) is an intermediate or borderline condition between optimal health and illness, characterised by the emergence of health issues, general weakness, and/or low energy [1]. Individuals with SHS experience declines in vitality, physiological function, and their capacity for adaptation, which often leads to higher incidence of chronic or infectious diseases [1]. As an emerging public health concern, SHS has been receiving growing attention from medical professionals [2]. However, due to inconsistencies in SHS measurements and the heterogeneity of targeted populations, reported rates of SHS vary from 20% to 80% [3,4]. There is currently no specific treatment for this condition, and patients suffering from it experience a reduced quality of life, have to frequently visit hospitals, and suffer additional non-medical expenses [5,6]. Several studies have shown that SHS is associated with the development of conditions such as diabetes mellitus, coronary heart disease, and stroke [7-10].

As a reversible stage preceding somatic or mental disorders, SHS is regarded as a lifestyle-related complaint associated with insufficient sleep, work- or study-related stress, physical inactivity, and an unhealthy diet [9,11,12]. According to previous studies, engaging in regular physical activity, maintaining a normal weight, quitting smoking, and abstaining from alcohol abuse can help minimise the risks of mortality from various chronic diseases and certain infectious diseases, with a particular focus on cardiovascular diseases and cancers [13,14]. Healthy lifestyles could enhance individuals’ ability to maintain their energy balance, resulting in optimal metabolic function and a significant reduction in the risk of chronic diseases [15,16]. Conversely, unhealthy lifestyles have the opposite effect and are widely recognised as modifiable risk factors in disease prevention and management [17,18], having reached the scale of an epidemic and showing the potential to burden society by way of an increasing prevalence of chronic adult diseases in the future [19].

Chronic diseases develop through a long-term process which lasts several years following exposure to unhealthy lifestyles. Exploring related lifestyles may help prevent chronic diseases during their early phases. However, studies have shown that community residents in China adopt healthy lifestyles at a low or moderate level [20]. Additionally, little is known about the current SHS based on large-scale representative national samples in China, despite individual-level studies involving diverse populations, including college students, teachers, civil servants, businessmen, medical personnel, and general community residents [12]. To address this gap, we conducted a nationwide cross-sectional survey to explore the prevalence of SHS among Chinese residents and identify the influencing lifestyle factors.

## METHODS

### Study design and participants

We conducted a national population-based cross-sectional survey of 148 cities across China [21]. We initially determined sampling proportions based on population distribution data from the Seventh National Census Data of China, encompassing 23 provinces, five autonomous regions, and four municipalities, after which we used a multistage random sampling approach at the provincial, municipal, district/county, and community/village levels to gather our study sample.

We included permanent residents with Chinese nationality who were ≥16 years old and had sufficient ability to read and understand the questionnaires. We excluded individuals currently diagnosed with somatic diseases or psychiatric abnormalities, those with a history of psychiatric abnormalities, those who had taken medication within the past two weeks, and those currently involved in other clinical investigations. We also excluded questionnaires that had been completed in less than 240 seconds during the interviews, if they had inconsistent logic within the responses or incomplete information, and if there were duplicates.

We publicly recruited the interviewers from local universities in each city; they received training in sampling methods, research tools, and quality control, and their skills were subsequently assessed with a predefined training protocol. The Shaanxi Health Culture Research Center ethics review board approved the protocol, which we also registered in the Chinese Clinical Trial Registry (ChiCTR2200061046). All participants have signed the informed consent form.

### Measurements of demographic characteristics and lifestyles

We collected data on the participants’ age, gender, body mass index (BMI), educational level, marital status, living area and household income per capita. Following the Chinese criteria of BMI [22], we categorised participants as thin (BMI<18.5), normal (BMI = 18.5-23.99), overweight (BMI = 24-27.99) and obese (BMI≥28). Lifestyle behavioral variables were alcohol consumption, smoking status, breakfast habits, frequency of takeaway food consumption per week, intermittent fasting, sleep duration, and physical activity.

### Measurement of SHS

We measured SHS with the Short-Form Suboptimal Health Status Questionnaire (SHSQ-SF) [23], which had a Cronbach’s α coefficient of 0.902 and a split-half reliability of 0.863, as well as good validity and reliability per the Kaiser-Meyer-Olkin (KMO) and Bartlett’s tests. Following scoring using a Likert 5-point scale (range = 0-4), we calculated a cumulative SHS score, categorising participants as either being in optimal health (total score <11) or suboptimal health (total score ≥11) [23].

### Statistical analysis

We presented continuous variables as means and standard deviations for normally distributed or medians and interquartile ranges (IQRs) for non-normally distributed data, and categorical variables as rates or percentages. We used Pearson χ^2^ test to compare the differences in the prevalence of SHS between groups and applied multivariate logistic regression analysis to identify the association between SHS and lifestyles. We performed all analyses in SPSS, version 25.0 (IBM Corporation, Armonk, New York, USA) and R, version 4.2.1 (R Core Team, Vienna, Austria). Statistical significance was set at a two-tailed *P* < 0.05.

## RESULTS

### Characteristic of study participants

**Figure 1:**
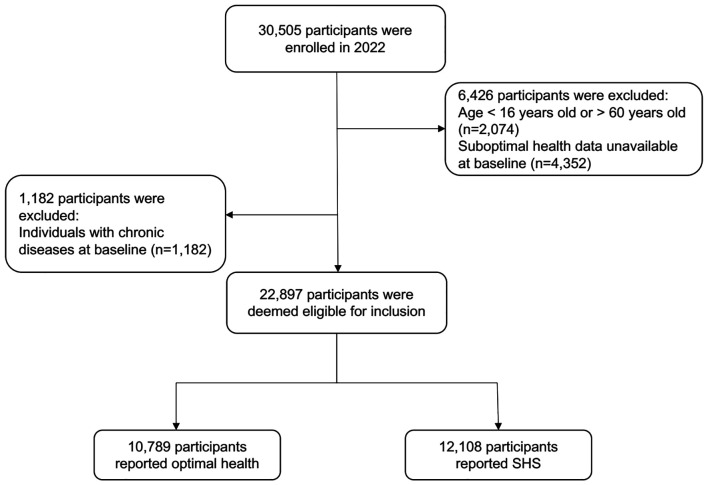
Flowchart of participant recruitment.

We excluded 7608 respondents with incomplete data (n = 6426) or the presence of somatic or psychological disorders (n = 1182) (**Figure 1**), resulting in a final sample of 22 897 participants (female: 13 056, male: 9841), representing 23 provinces, five autonomous regions, and four municipalities across China. We recruited 5662 (24.73%) participants from rural and 17 235 (75.27%) from urban areas. Widowed or divorced participants comprised 1.85% of the sample, while smokers and drinkers were 14.55% and 31.45%, respectively (**Table 1**).

**Table 1:** Characteristics of study participants

Variables, n (%)	OHS (n = 10 789)	SHS (n = 12 108)	Total (n = 22 897)	χ^2^	*P*-value
**Age group in years**				215.67	<0.001
16-25	4795 (42.73)	6426 (57.27)	11 221 (49.01)		
26-35	2025 (50.73)	1967 (49.27)	3992 (17.43)		
36-45	1726 (50.06)	1722 (49.94)	3448 (15.06)		
46-55	1785 (50.8)	1729 (49.2)	3514 (15.35)		
56-65	458 (63.43)	264 (36.57)	722 (3.15)		
**Gender**				79.76	<0.001
Male	4971 (50.51)	4870 (49.49)	9841 (42.98)		
Female	5818 (44.56)	7238 (55.44)	13 056 (57.02)		
**Marital status**				208.49	<0.001
Unmarried	5531 (43.13)	7292 (56.87)	12 823 (56.00)		
Married	5085 (52.69)	4566 (47.31)	9651 (42.15)		
Divorced	134 (40.73)	195 (59.27)	329 (1.44)		
Widowed	39 (41.49)	55 (58.51)	94 (0.41)		
**Living area**				0.18	>0.05
Urban	8135 (47.2)	9100 (52.8)	17 235 (75.27)		
Rural	2654 (46.87)	3008 (53.13)	5662 (24.73)		
**BMI**				52.82	<0.001
Underweight (<18.5)	1578 (41.93)	2185 (58.07)	3763 (16.43)		
Normal (18.5-24)	6805 (48.3)	7285 (51.7)	14 090 (61.54)		
Overweight (24-28)	1945 (48.38)	2075 (51.62)	4020 (17.56)		
Obese (≥28)	461 (45.02)	563 (54.98)	1024 (4.47)		
**Income per capita in CNY/mo**				58.78	<0.001
≤3000	3191 (43.9)	4078 (56.1)	7269 (31.75)		
3001-5999	4571 (49.12)	4734 (50.88)	9305 (40.64)		
6000-8999	1380 (50.36)	1360 (49.64)	2740 (11.97)		
9000-11 999	747 (46.08)	874 (53.92)	1621 (7.08)		
≥12 000	900 (45.87)	1062 (54.13)	1962 (8.56)		
**Education level**				160.56	<0.001
Primary school or below	659 (55.85)	521 (44.15)	1180 (5.15)		
Middle school	1313 (56.79)	999 (43.21)	2312 (10.10)		
High school	2764 (47.52)	3052 (52.48)	5816 (25.41)		
University/college	5630 (44.42)	7044 (55.58)	12 674 (55.35)		
Graduate school (e.g. master’s/doctoral degree)	423 (46.23)	492 (53.77)	915 (3.99)		

BMI – body mass index, CNY – Chinese Yuan, SHS – suboptimal health status, OHS – optimal health status, mo – month

### The prevalence of SHS

The prevalence of SHS was 52.88% overall and was significantly higher among women (55.44%) than men (49.49%) (*P* < 0.001). We found no statistically significant differences in prevalence between urban (52.80%) and rural participants (53.13%) (*P* > 0.05). Meanwhile unmarried ones had a higher prevalence (56.87%) than married ones (47.31%) (*P* < 0.001). Participants classified as underweight had the highest prevalence of SHS (58.07%) compared to other categories (*P* < 0.001).

Prevalence rates were the highest for participants who stopped smoking (68.28%), followed by those who currently smoked (56.49%) and those who never smoked (51.93%) (*P* < 0.001) (**Table 2**). Similarly, those who stopped drinking alcohol (62.42%) had the highest rates, followed by those who currently drank (60.12%) and those who never drank it (49.27%) (*P* < 0.001). Participants who fasted intermittently had the higher prevalence rates (69.79%) than those who did not (50.84%) (*P* < 0.001).

**Table 2:** Descriptive results of SHS-related lifestyles

Variables, n (%)	OHS (n = 10 789)	SHS (n = 12 108)	Total (n = 22 897)	χ^2^	*P*-value
**Smoking**				74.73	<0.001
Never smoking	9406 (48.07)	10 161 (51.93)	19 567 (85.85)		
Ceased smoking	177 (31.72)	381 (68.28)	558 (2.44)		
Currently smoking	1206 (43.51)	1566 (56.49)	2772 (12.11)		
**Alcohol consumption**				264.54	<0.001
Never drinking	7963 (50.73)	7733 (49.27)	15 696 (68.55)		
Ceased drinking	752 (37.58)	1249 (62.42)	2001 (8.74)		
Currently drinking	2074 (39.88)	3126 (60.12)	5200 (22.71)		
**Frequency breakfast per week**				533.72	<0.001
≤2 times	1612 (36.47)	2808 (63.53)	4420 (19.30)		
3-4 times	1425 (38.49)	2277 (61.51)	3702 (16.17)		
5-6 times	1433 (46.66)	1638 (53.34)	3071 (13.41)		
7 times	6319 (53.99)	5385 (46.01)	11 704 (51.12)		
**Number of takeaways per week**				330.45	<0.001
0 time	1604 (38.5)	2562 (61.5)	4166 (18.19)		
1 time	1896 (59.6)	1285 (40.4)	3181 (13.89)		
2 times	3853 (47.89)	4192 (52.11)	8045 (35.14)		
≥3 times	3436 (45.78)	4069 (54.22)	7505 (32.78)		
**Intermittent fasting**				316.82	<0.001
Yes	744 (30.21)	1719 (69.79)	2463 (10.76)		
No	10 045 (49.16)	10 389 (50.84)	20 434 (89.24)		
**Sleep duration per day**				691.30	<0.001
≤5h	399 (31.87)	853 (68.13)	1252 (5.47)		
6h	1568 (34.12)	3028 (65.88)	4596 (20.07)		
7h	4363 (47.9)	4746 (52.1)	9109 (39.78)		
>7h	4459 (56.16)	3481 (43.84)	7940 (34.68)		
**Vigorous-intensity activity**				75.86	<0.001
Occasionally	8299 (47.06)	9337 (52.94)	17 636 (77.02)		
Sometimes	1347 (44.46)	1683 (55.54)	3030 (13.24)		
Often	544 (44.34)	683 (55.66)	1227 (5.36)		
Everyday	599 (59.66)	405 (40.34)	1004 (4.38)		
**Mild-intensity activity**				71.43	<0.001
Occasionally	1918 (40.02)	2875 (59.98)	4793 (20.93)		
Sometimes	1782 (41.32)	2531 (58.68)	4313 (18.83)		
Often	1717 (45.74)	2037 (54.26)	3754 (16.40)		
Everyday	5372 (53.52)	4665 (46.48)	10 037 (43.84)		

SHS – suboptimal health status, OHS – optimal health status, h – hours

### Association between demographic information and SHS

The multivariate regression analysis indicated that women (OR = 1.514; 95% CI = 1.422-1.612) and individuals living in rural areas (OR = 1.094; 95% CI = 1.023-1.171) had a higher risk of SHS. Conversely, being married (OR = 0.858; 95% CI = 0.770-0.956) and having a normal BMI (OR = 0.863; 95% CI = 0.799-0.932) had a protective effect on health status. Additionally, income and age were found to have potential impacts on the development of SHS (**Table 3**).

**Table 3:** Logistic regression analysis of SHS-related lifestyles

	Univariate model		Multivariate model	
**Variables**	**OR (95% CI)**	***P*-value**	**OR (95% CI)**	***P*-value**
**Smoking**				
Never smoking	ref		ref	
Ceased smoking	1.993 (1.664-2.387)	<0.001	1.959 (1.613-2.380)	<0.001
Currently smoking	1.202 (1.109-1.302)	<0.001	1.165 (1.058-1.283)	<0.01
**Alcohol consumption**				
Never drinking	ref		ref	
Ceased drinking	1.710 (1.554-1.882)	<0.001	1.641 (1.479-1.820)	<0.001
Currently drinking	1.552 (1.456-1.654)	<0.001	1.483 (1.377-1.596)	<0.001
**Frequency of breakfast per week**				
≤2 times	ref		ref	
3-4 times	0.917 (0.838-1.004)	>0.05	0.910 (0.827-1.001)	>0.05
5-6 times	0.656 (0.598-0.721)	<0.001	0.702 (0.636-0.776)	<0.001
7 times	0.489 (0.456-0.525)	<0.001	0.617 (0.570-0.667)	<0.001
**Number of takeaways per week**				
0 time	ref		ref	
1 time	1.605 (1.477-1.745)	<0.001	1.367 (1.251-1.494)	<0.001
2 times	1.747 (1.606-1.901)	<0.001	1.426 (1.299-1.565)	<0.001
≥3 times	2.357 (2.144-2.590)	<0.001	1.781 (1.604-1.979)	<0.001
**Intermittent fasting**				
No	ref		ref	
Yes	2.234 (2.041-2.445)	<0.001	1.629 (1.481-1.793)	<0.001
**Sleep duration per day**				
>7h	ref		ref	
7h	1.393 (1.312-1.480)	<0.001	1.426 (1.339-1.520)	<0.001
6h	2.474 (2.294-2.667)	<0.001	2.336 (2.159-2.528)	<0.001
≤5h	2.738 (2.412-3.109)	<0.001	2.207 (1.931-2.522)	<0.001
**Vigorous-intensity activity**				
Occasionally	ref		ref	
Sometimes	1.111 (1.028-1.200)	<0.01	1.086 (1.000-1.179)	>0.05
Often	1.116 (0.993-1.254)	>0.05	1.097 (0.970-1.241)	>0.05
Everyday	0.601 (0.528-0.684)	<0.001	0.724 (0.631-0.832)	<0.001
**Mild-intensity activity**				
Occasionally	ref		ref	
Sometimes	0.948 (0.871-1.030)	>0.05	0.969 (0.887-1.059)	>0.05
Often	0.791 (0.726-0.863)	<0.001	0.857 (0.781-0.939)	<0.01
Everyday	0.579 (0.540-0.621)	<0.001	0.683 (0.633-0.737)	<0.001
**Age group in years**				
16-25	ref		ref	
26-35	0.725 (0.674-0.779)	<0.001	0.850 (0.769-0.941)	<0.01
36-45	0.744 (0.690-0.804)	<0.001	0.983 (0.865-1.117)	>0.05
46-55	0.723 (0.670-0.780)	<0.001	1.035 (0.906-1.181)	>0.05
56-65	0.430 (0.368-0.503)	<0.001	0.680 (0.559-0.827)	<0.001
**Gender**				
Male	ref		ref	
Female	1.270 (1.205-1.338)	<0.001	1.514 (1.422-1.612)	<0.001
**Marital status**				
Unmarried	ref		ref	
Married	0.681 (0.646-0.718)	<0.001	0.858 (0.770-0.956)	<0.01
Divorced	1.104 (0.883-1.379)	>0.05	1.032 (0.802-1.330)	>0.05
Widowed	1.070 (0.709-1.615)	>0.05	1.221 (0.780-1.910)	>0.05
**Living area**				
Urban	ref		ref	
Rural	1.013 (0.954-1.076)	>0.05	1.094 (1.023-1.171)	<0.01
**BMI**				
Underweight (<18.5)	ref		ref	
Normal (18.5-24)	0.773 (0.719-0.831)	<0.001	0.863 (0.799-0.932)	<0.001
Overweight (24-28)	0.770 (0.704-0.843)	<0.001	0.935 (0.847-1.032)	>0.05
Obese (≥28)	0.882 (0.767-1.014)	>0.05	0.992 (0.854-1.152)	>0.05
**Income per capita in CNY/mo**				
≤3000	ref		ref	
≤3000	0.810 (0.762-0.862)	<0.001	0.834 (0.780-0.892)	<0.001
3001-5999	0.771 (0.706-0.842)	<0.001	0.757 (0.687-0.833)	<0.001
6000-8999	0.916 (0.822-1.020)	>0.05	0.883 (0.786-0.992)	<0.05
9000-11 999	0.923 (0.835-1.021)	>0.05	0.846 (0.758-0.945)	<0.01
**Education level**				
Primary school or below	ref		ref	
Middle school	0.962 (0.836-1.108)	>0.05	0.873 (0.751-1.015)	>0.05
High school	1.397 (1.231-1.584)	<0.001	1.068 (0.927-1.230)	>0.05
University/college	1.583 (1.403-1.785)	<0.001	1.135 (0.989-1.303)	>0.05
Graduate school (e.g. master’s/doctoral degree)	1.471 (1.237-1.750)	<0.001	1.203 (0.990-1.461)	>0.05

BMI – body mass index, CNY – Chinese Yuan, SHS – suboptimal health status, OHS – optimal health status, CI – confidence interval, OR – odds ratio, ref – reference, h – hours, mo – months

### Association between Lifestyles and SHS

**Figure 2:**
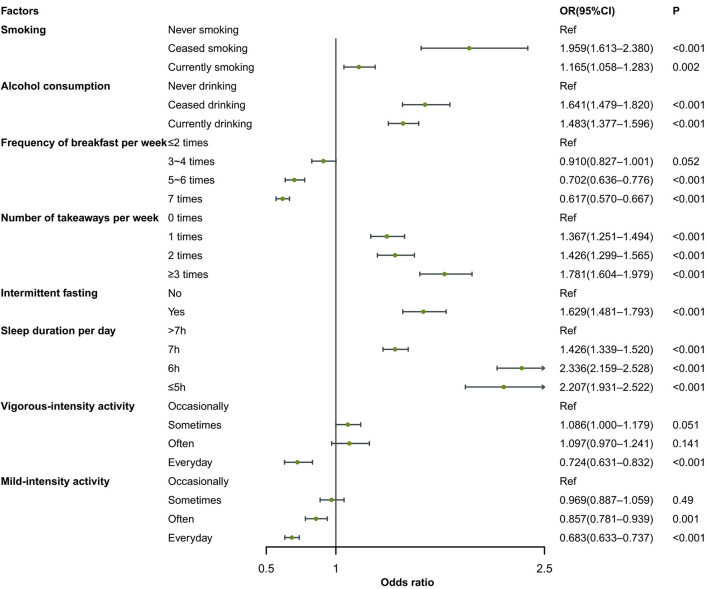
Forest map of the multivariate logistic regression analysis. CI – confidence interval, OR – odds ratio, ref – reference.

After adjusting for age, gender, marital status, living area, household income, BMI, and education level, ceasing to smoke (OR = 1.959; 95% CI = 1.613-2.380) and current smoking (OR = 1.165; 95% CI = 1.058-1.283) compared to never smoking, as well as ceasing to drinking alcohol (OR = 1.641; 95% CI = 1.479-1.820) and current drinking (OR = 1.483; 95% CI = 1.377-1.596) compared to never drinking alcohol emerged as risk factors for SHS, as did frequency of takeaways per week (one time: OR = 1.357, two times: OR = 1.426, three or more times: OR = 1.781), intermittent fasting (OR = 1.629; 95% CI = 1.481-1.793), and shorter sleep duration (five or less hours: OR = 2.207, six hours: OR = 2.336, six to seven hours: OR = 1.426) compared to sleep duration more than seven hours (**Figure 2** and **Table 3**).

Inversely, higher frequency of breakfast per week (five to six times: OR = 0.702, seven times: OR = 0.617) compared with two or fewer times per week and more frequent mild-intensity activity (often: OR = 0.857; 95% CI = 0.781-0.939, every day: OR = 0.683; 95% CI = 0.633-0.737) compared with occasional activity emerged as protective factors.

## DISCUSSION

**Figure 3:**
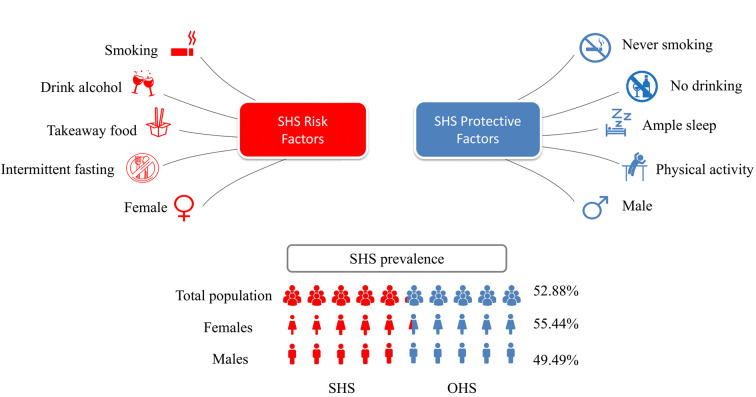
Risk and protective factors of SHS. SHS – suboptimal health status, OHS – optimal health status.

Approximately 52.88% of the 22 897 participants aged 16-65 years in our nationwide cross-sectional study reported complaints related to SHS. Individuals exposed to unhealthy dietary habits, exercising insufficiently, smoking, drinking exercise, and sleeping less had an increased risk of SHS (**Figure 3**).

Early identification of SHS and its associated determinants (e.g. eating and activity habits), along with targeted interventions, can play a crucial role in mitigating adverse outcomes and offer a comprehensive and holistic way to prevent chronic diseases and enhance the quality of life.

The consumption of takeaway food increased over the past decade, emerging as a significant factor contributing to the elevating incidence of overweight and/or obesity due to its unfavourable nutritional contents [24-26]. This diet habit, in turn, predisposes individuals to cardiovascular diseases (CVD) [27-29], type 2 diabetes [30,31], and cancers [25].

Moreover, a previous observational study found that unhealthy diet is associated with a higher likelihood of mental health problems in the adult population [32]. Reduced consumption of well-balanced nutrient-rich foods (e.g. a lack of fruits and vegetables) and/or higher consumption of energy-dense foods was shown to be independently associated with increased levels of stress and depression [32-37]. These mental health factors are key determinants of SHS.

Individuals with regular breakfast tend to have healthier overall lifestyle patterns, a greater focus on healthy nutritional patterns, improved relationships, and increased ability to self-manage stress [38]. Moreover, habitual breakfast eaters exhibit a higher proclivity to physical activities than those with irregular breakfast [38]. We had similar findings, demonstrating a significant positive association between the prevalence of SHS and irregular breakfast consumption and the consumption of takeaway food.

Intermittent fasting leads to changes in the body’s energy metabolism processes and affects the progression of various diseases. However, inconsistent with a previous study [39], we did not find it to be associated with lower risk of SHS. Moreover, the body tends to adapt to long-term regular meal times, so short-term intermittent fasting might lead to a perception of poor health, despite the long-term health benefits of more regular fasting. Physical activity and regular exercise have been shown to reduce the risk of NCDs in a dose-dependent manner, including cardiovascular diseases, type 2 diabetes, and cancers [40]. Our findings further confirm this and suggest that frequent mild-intensity physical activities are associated with a lower prevalence of SHS [41].

Sleep is an essential biological and behavioral process that significantly contributes to the maintenance of overall health and quality of life [42], acting as a modulator of cardiovascular function, blood glucose regulation, and hormonal secretion [43]. Insufficient sleep duration can impact metabolic regulation, autonomic nervous functions, blood coagulation system, and endothelial dysfunction [44]. We have observed that lack of sleep was associated with higher risk of SHS, which is in line with previous studies [45-47].

We also found a higher prevalence of SHS among smokers compared to those who have never smoked, while ex-smokers had a higher prevalence than either group. This phenomenon may be attributed to the common issue of reverse causality often encountered in cross-sectional studies, where individuals who have experienced health problems may decide to quit smoking. This is similar to the rationale behind the observation that individuals who have ceased alcohol drinking had a higher prevalence of SHS than current drinkers. Furthermore, long-term smoking and drinking often result in the body adapting to these behaviors. Therefore, individuals’ cessation of these habits can disturb the body’s equilibrium and potentially contribute to a perception of declining health [48].

### Limitations

We designed this study as an observational, cross-sectional survey; this allowed us to explore the national prevalence of SHS and the association between lifestyle factors and SHS, but prevented us from establishing causality, which should be the purpose of future longitudinal studies. Second, we did not define prevalence of SHS based on sex-specific cut-off points. Third, we obtained all information through self-reported questionnaires, which may introduce potential information bias. Fourthly, although we assessed SHS using a standardised questionnaire, methodological differences may exist when compared to studies conducted in other populations or employing different questionnaires. Lastly, we assessed the frequency of consuming takeaway food per week, yet the term “takeaway” encompasses a broad range of foods, some of which may be healthy, while others are not, resulting in varying effects on consumers’ health.

## CONCLUSIONS

We found that unhealthy dietary habits, insufficient exercise, smoking, alcohol drinking, and short sleep duration were associated with an increased risk of SHS, underscoring the importance of adopting healthy lifestyles to enhance health status and prevent chronic diseases.
